# Conjugation of ATG8s to single membranes at a glance

**DOI:** 10.1242/jcs.261031

**Published:** 2024-08-15

**Authors:** Carmen Figueras-Novoa, Lewis Timimi, Elena Marcassa, Rachel Ulferts, Rupert Beale

**Affiliations:** ^1^ Cell Biology of Infection Laboratory, The Francis Crick Institute, London NW1 1AT, UK; ^2^ Division of Medicine, University College London, London NW1 1AT, UK

**Keywords:** Autophagy, TECPR1, V-ATPase, CASM, STIL, VAIL, ATG8ylation

## Abstract

Autophagy refers to a set of degradative mechanisms whereby cytoplasmic contents are targeted to the lysosome. This is best described for macroautophagy, where a double-membrane compartment (autophagosome) is generated to engulf cytoplasmic contents. Autophagosomes are decorated with ubiquitin-like ATG8 molecules (ATG8s), which are recruited through covalent lipidation, catalysed by the E3-ligase-like ATG16L1 complex. LC3 proteins are ATG8 family members that are often used as a marker for autophagosomes. In contrast to canonical macroautophagy, conjugation of ATG8s to single membranes (CASM) describes a group of non-canonical autophagy processes in which ATG8s are targeted to pre-existing single-membrane compartments. CASM occurs in response to disrupted intracellular pH gradients, when the V-ATPase proton pump recruits ATG16L1 in a process called V-ATPase–ATG16L1-induced LC3 lipidation (VAIL). Recent work has demonstrated a parallel, alternative axis for CASM induction, triggered when the membrane recruitment factor TECPR1 recognises sphingomyelin exposed on the cytosolic face of a membrane and forms an alternative E3-ligase-like complex. This sphingomyelin–TECPR1-induced LC3 lipidation (STIL) is independent of the V-ATPase and ATG16L1. In light of these discoveries, this Cell Science at a Glance article summarises these two mechanisms of CASM to highlight how they differ from canonical macroautophagy, and from each other.

## Teaser

**Summary:** A description of the distinct pathways and mechanisms of what is sometimes called ‘non-canonical autophagy’, highlighting the use of terms such as CASM, VAIL and STIL for ATG8 targeting of membranes in different contexts.

**Figure:**
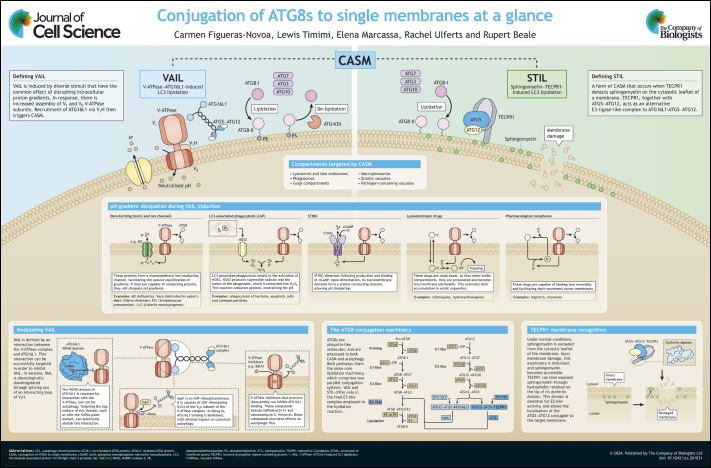
See Supplementary information for a high-resolution version of the poster.

## Introduction

Membrane-bound compartments are required for maintaining electrochemical gradients within cells. This underpins organelle specialisation and is essential for eukaryotic life. Understanding how the cell addresses threats to compartmental integrity is a central question in biology. Macroautophagy (see Glossary) is a highly conserved degradative pathway that has central roles in compartmental homeostasis and host defence (Aman et al., 2021; Miller and Thorburn, 2021). It is referred to interchangeably by the shorthand ‘autophagy’, although other pathways for lysosomal degradation of cytoplasmic contents are known (Yim and Mizushima, 2020). In (macro)autophagy, double-membraned structures, termed autophagosomes, are generated *de novo* to engulf cytoplasmic cargo, including damaged organelles, invading microorganisms and misfolded or aggregated proteins (Vargas et al., 2023). The autophagosome then fuses with the lysosome to form the autophagolysosome, where its contents are degraded (Hurley and Young, 2017; Yu et al., 2018). Key players in this process are the ATG8 family of proteins (ATG8s, see Glossary), which in humans includes six active genes that are divided into the microtubule-associated protein 1 light chain 3 (LC3) and γ-aminobutyric acid receptor-associated protein (GABARAP) subfamilies (Shpilka et al., 2011). During autophagy, ATG8s undergo covalent conjugation (lipidation, denoted ATG8-I when non-lipidated and ATG8-II when lipidated) to phospholipids, classically phosphatidylethanolamine (PE), within the membrane of the forming autophagosome (Ichimura et al., 2000; Noda et al., 2009; Durgan et al., 2021). ATG8 lipidation is a key step in autophagosome maturation and cargo selection (Lystad and Simonsen, 2019; Nieto-Torres et al., 2021).

**Glossary**

**ATG8s:** ubiquitin-like molecules that can become covalently conjugated to phospholipids (lipidated) and thus targeted to membranes.

**ATG8ylation:** this describes the attachment of ATG8s by lipidation to any target structure, usually a membrane. This can be in the context of macroautophagy or CASM.

**CASM:** conjugation of ATG8s to single membranes. This is a descriptive term referring to ATG8s becoming lipidated at pre-existing, single-membrane structures such as endosomes or lysosomes.

**LAP:** LC3-associated phagocytosis. This is a form of VAIL that takes place when phagosomal compartments cannot be acidified, usually due to superoxide production.

**Macroautophagy:** refers to the engulfment of cytoplasmic contents by a double-membraned autophagosome and its targeting to the lysosome (or in yeasts, the vacuole) for degradation. Often just referred to as autophagy.

**‘Non-canonical autophagy’:** sometimes used to describe any process that is related to macroautophagy or shares some of the same machinery. We suggest instead using the terms CASM when it is clear ATG8s are targeted to single membranes. VAIL or STIL can be used when the mechanism is known. ATG8ylation can be used when the target and mechanism is unclear.

**STIL:** sphingomyelin–TECPR1-induced LC3 lipidation. This refers to ATG8s becoming targeted to membranes when sphingomyelin is abnormally distributed on the cytosolic-facing leaflet of a membrane. This is recognised by tectonin β-propeller repeat containing 1 (TECPR1), which forms an alternative E3-ligase-like complex for ATG8 lipidation.

**VAIL:** V-ATPase–ATG16L1-induced LC3 lipidation. This refers to ATG8s becoming targeted to membranes due to recruitment of the ATG16L1 complex by the vacuolar-type ATPase (V-ATPase) when pH gradients cannot be maintained.

ATG8s are similar to ubiquitin in structure, and undergo lipidation via a ubiquitin-like E1, E2, E3 conjugation reaction (for a detailed review, see Lystad and Simonsen, 2019). The final E3-like lipidation step is catalysed by a complex formed between a covalent conjugate of ATG5–ATG12 and an additional protein that determines the target membrane (see poster). A membrane that has been covalently targeted by ATG8s is said to be ATG8ylated (Deretic and Lazarou, 2022; see Glossary). Although several proteins form active complexes with ATG5–ATG12, only ATG16L1 drives ATG8 lipidation in the context of macroautophagy (Mizushima et al., 1999; Fujita et al., 2008). Recruitment of this ATG16L1-ATG5–ATG12 complex to autophagosomal membranes involves binding of this complex to FAK family-interacting protein of 200 kDa (FIP200, also known as RB1CC1) and WD repeat domain, phosphoinositide interacting 2 (WIPI2) via binding sites located in the coiled-coil region of ATG16L1 (Gammoh et al., 2013; Dooley et al., 2014).

ATG8 lipidation also occurs in pathways distinct from macroautophagy (Nieto-Torres et al., 2021; Durgan and Florey, 2022). Here, pre-existing single-membrane compartments – rather than newly forming autophagosomes – are targeted in a process known as ‘conjugation of ATG8s to single membranes’ (CASM) (see Glossary and poster) (Durgan and Florey, 2022; Wang et al., 2022). This process, like macroautophagy, involves ATG8 conjugation to PE, but a small proportion of ATG8 can also be conjugated to phosphatidylserine (PS) during CASM (Durgan et al., 2021). A variety of single-membrane organelles can be targeted by CASM, including those of the endo-lysosomal system (Florey et al., 2015; Jacquin et al., 2017), Golgi compartments (Gao et al., 2016; Liu et al., 2023) and phagosomes [termed LC3-associated phagocytosis (LAP); see Glossary] (Sanjuan et al., 2007; Fletcher et al., 2018; Hooper et al., 2022). The precise cell biological processes downstream of CASM induction are still not fully elucidated.

CASM was originally thought to exclusively occur via one mechanism, known as V-ATPase–ATG16L1-induced LC3 lipidation (VAIL; see Glossary) (Xu et al., 2019; Fischer et al., 2020; Ulferts et al., 2021; Hooper et al., 2022). This process also involves the ATG16L1-ATG5–ATG12 complex but is distinguishable from autophagy by the mechanism of ATG16L1 recruitment. The vacuolar-type ATPase (V-ATPase), a transmembrane proton pump essential for acidification of endolysosomal and secretory compartments, regulates VAIL. Initially, co-immunoprecipitation experiments suggested an inducible interaction between ATG16L1 and the V-ATPase (Xu et al., 2019; Fischer et al., 2020; Ulferts et al., 2021). More recently, we have demonstrated a direct interaction between the V_1_H subunit of the V-ATPase and ATG16L1 (Timimi et al., 2024).

The terms VAIL and CASM have been used interchangeably under the assumption that CASM always proceeds through this axis. However, an ATG16L1-independent form of CASM has recently been described (Boyle et al., 2023; Corkery et al., 2023; Kaur et al., 2023). In this alternative pathway, tectonin β-propeller repeat containing 1 (TECPR1) forms a complex with ATG5–ATG12 and induces lipidation of ATG8s to single-membrane structures in the absence of ATG16L1 (see poster). Unlike VAIL, this pathway depends on the exposure of sphingomyelin in damaged membranes (Boyle et al., 2023; Corkery et al., 2023; Kaur et al., 2023), and we therefore suggest sphingomyelin–TECPR1-induced LC3 lipidation (STIL; see Glossary) could be used to describe this pathway.

In this Cell Science at a Glance article, we discuss recent discoveries relating to CASM, highlighting the mechanisms of VAIL and STIL and how they differ from one another and from autophagy. We suggest that CASM should be used as a descriptive term encompassing any ATG8 conjugation event at a single-membrane compartment, and hope this article encourages the use of more precise terminology in the field (see Glossary).

## VAIL mechanisms

The V-ATPase is a proton pump that is required to establish acidic pH in the endo-lysosomal and secretory compartments (Freeman et al., 2022). It is a molecular machine composed of the catalytic V_1_ and transmembrane V_O_ subcomplexes that reversibly associate to regulate its activity (Oot et al., 2017; Vasanthakumar and Rubinstein, 2020; Vasanthakumar et al., 2022). Following pH gradient dissipation, V_1_V_O_ association increases, and this coincides with an increase in ATG16L1 recruitment (Hooper et al., 2022). We have recently discovered a mechanism by which ATG16L1 recruitment occurs: the V_1_H component of the catalytic V_1_ subcomplex is only accessible to ATG16L1 binding within the fully assembled V_1_V_O_ proton pump, and is not when present in the dissociated V_1_ complexes. Thus, an increase in V_1_V_O_ association at a single-membrane compartment is capable of driving ATG16L1 recruitment (Timimi et al., 2024).

ATG16L1 contains three main domains: an N-terminal ATG5-binding domain, a coiled-coil domain (CCD) and a C-terminal WD40 domain. The WD40 domain is required for VAIL (Fletcher et al., 2018); deletion of this domain or point mutation of key residues abolishes VAIL by disrupting the V-ATPase–ATG16L1 interaction (Xu et al., 2019; Ulferts et al., 2021; Timimi et al., 2024). However, the WD40 domain alone is not sufficient to bind the V-ATPase, and additional contributions from the coiled-coil domain are required (Xu et al., 2019). During VAIL, the V-ATPase recruits the ATG16L1-ATG5–ATG12 complex to single-membrane compartments, bypassing the requirement for upstream autophagic initiator complexes, such as the Unc-51-like kinase (ULK) complex, phosphatidylinositol 3-kinase class III (PIK3C3) and phosphatidylinositol-3-phosphate (PI3P) effectors, such as WIPI2 (Xu et al., 2019; Ulferts et al., 2021; Hooper et al., 2022). Consistent with independent regulation of VAIL, the WD40 domain of ATG16L1 is not required for ATG8 lipidation during macroautophagy. Reciprocally, it has been shown that binding sites on ATG16L1 for canonical autophagic regulators, such as FIP200, are dispensable for VAIL (Fletcher et al., 2018).

## VAIL induction

Diverse stimuli have been described to induce VAIL, from pathogens to pharmacological agents (Xu et al., 2019; Ulferts et al., 2021; Hooper et al., 2022). These include the viroporin matrix 2 (M2) of influenza A virus (IAV) (Beale et al., 2014; Fletcher et al., 2018), pore-forming toxins (PFTs) of bacteria (e.g. VacA of *Helicobacter pylori*) (Florey et al., 2015), lysosomotropic drugs and ionophores (Florey et al., 2015; Jacquin et al., 2017), and certain types of endocytosis (Sanjuan et al., 2007; Florey et al., 2011; Heckmann et al., 2019). Despite this diversity, VAIL inducers have a common effect of disrupting the pH of organelles that would usually be acidic (Durgan and Florey, 2022).

### Pathogen-induced VAIL

VAIL has been observed in response to intracellular infections (Wang et al., 2022). Many pathogens modulate intracellular pH to regulate maturation of pathogen-encoded proteins or to create a permissible environment for replication (Westman and Grinstein, 2020). These pathogen-driven changes in host pH homeostasis can be detected via VAIL.

Viroporins are viral ion channels, many of which conduct cations with varying degrees of selectivity (Nieva et al., 2012). For example, M2 from IAV is a highly selective proton-conducting channel (Takeuchi and Lamb, 1994). M2 neutralises the pH of the secretory pathway during viral assembly and egress. This is required by IAV to prevent premature activation of haemagglutinin (HA), which undergoes a conformational change in the acidic pH of endosomes in a target cell to allow entry of IAV (Skehel et al., 1982; Bullough et al., 1994; Harrison, 2008). Without M2, HA can be prematurely triggered in the acidic pH of the trans-Golgi network and other post-Golgi compartments (Ciampor et al., 1992; Alvarado-Facundo et al., 2015). Through its effect on intracellular proton gradients, M2 can act as a potent inducer of VAIL (Beale et al., 2014; Fletcher et al., 2018). Because M2 is highly selective for protons, these observations were the among the first indications that disrupted pH gradients would be sensed by VAIL.

PFTs are expressed in both gram-negative and gram-positive bacteria. When inserted into membranes, PFTs form a hydrophilic core within the membrane, allowing the conduction of ions, including protons (Ulhuq and Mariano, 2022). Many PFTs have been described to induce VAIL, including MakA (from *Vibrio cholerae*) (Dongre et al., 2018; Corkery et al., 2021; Jia et al., 2022), pneumolysin (PLY, from *Streptococcus pneumoniae*) (Vögele et al., 2019; Inomata et al., 2020), listeriolysin O (LLO, from *Listeria monocytogeneses* (Gluschko et al., 2018, 2022) and VacA (from *Helicobacter pylori*) (see poster) (Genisset et al., 2007; Florey et al., 2015).

### LC3-associated phagocytosis

LAP is a distinctive form of VAIL (Wang et al., 2022) and is initiated by large extracellular cargo, including pathogens, such as *Mycobacterium tuberculosis* and *L. monocytogenes*, apoptotic cells and outer membrane vesicles (Florey et al., 2011; Chu et al., 2016; Wang et al., 2022; Yuan et al., 2022) (see poster). Although the downstream molecular mechanisms are still not fully understood, it appears that rather than being mediated by an increase in proton conductance across the membrane, LAP is mediated by the production of luminal-alkalising species within maturing phagosomes, such as the production of reactive oxygen species (ROS), primarily by NADPH oxidase 2 (NOX2) (Hooper et al., 2022). These oxygen species react with protons, resulting in alkalinisation of the lumen of organelles. This leads to increased V-ATPase assembly and ATG16L1 recruitment (Hooper et al., 2022).

### STING-induced VAIL

Stimulator of interferon genes (STING; also known as STING1) initiates type I interferon signalling following detection of double-strand DNA (dsDNA) in the cytosol (often a result of viral or bacterial infection) (Chen et al., 2016). Here, the dsDNA binds to and activates cyclic guanosine monophosphate (GMP)-adenosine monophosphate (AMP) synthase (cGAS). cGAS synthesises the cyclic dinucleotide 2′3′-cGMP-AMP (cGAMP), which in turn activates STING. In addition to type I interferon signalling, this also induces VAIL (Gui et al., 2019; Fischer et al., 2020) (see poster). Inactive STING resides in the endoplasmic reticulum (ER) membrane and once activated, is transported to the Golgi. STING was recently shown to act as a proton channel, inducing an increase in Golgi pH. Pharmacological inhibition of this Golgi proton leakage does not affect STING-dependent type I interferon expression, but is sufficient to inhibit downstream VAIL (Liu et al., 2023). Thus, these two functions of STING can be distinguished, suggesting that interferon signalling cascades are activated in parallel to VAIL, rather than downstream. Although a recent report indicates that VAIL is important for the localisation of ubiquitin foci after STING activation (Fischer et al., 2023 preprint), the broader role of VAIL in STING signalling remains unclear.

### Pharmacological compounds as inducers of VAIL

Numerous pharmacological agents have been shown to induce VAIL, including ionophores, which reversibly bind ions and catalyse their transport across hydrophobic membranes. Nigericin and monensin are ionophores that co-transport protons with a preference for K^+^ and Na^+^, respectively, and thus raise the pH of acidic organelles and induce VAIL (Tapper and Sundler, 1990; Florey et al., 2015; Jacquin et al., 2017). Alternatively, lysosomotropic drugs, such as the weakly basic chloroquine, accumulate in lysosomes, raising lysosomal pH and inducing VAIL (de Duve et al., 1974; Jacquin et al., 2017). Importantly, many lysosomotropic agents are employed in studies of canonical autophagy, where they lead to an increase in lipidated ATG8s through the inhibition of autophagosome degradation (Yoon et al., 2010; Jacquin et al., 2017; Klionsky et al., 2021). Given the effects on both VAIL and autophagy, studies of ATG8 lipidation using lysosomotropic drugs require careful and cautious interpretation.

## Modulating VAIL

VAIL inhibition can be accomplished by targeting the V-ATPase complex. Although this is currently primarily used in the laboratory to study the mechanism and physiological functions of VAIL, manipulation of VAIL could foreseeably become important in a therapeutic setting. VAIL ablation can be achieved pharmacologically with macrolide antibiotics like bafilomycin A1 and concanamycin A, which inhibit the V-ATPase complex and V-ATPase-mediated ATG16L1 recruitment (Florey et al., 2015; Jacquin et al., 2017). Inhibition of V-ATPase proton pumping has diverse cellular effects, including de-acidification of lysosomes. This leads to a block in macroautophagic flux in the presence of V-ATPase inhibitors, meaning VAIL-specific effects on ATG8 lipidation are difficult to ascertain without stringent controls. It is also important to note that some V-ATPase inhibitors can have the opposite, that is, an activating, effect on VAIL. For example, saliphenylhalamide is a pharmacological V-ATPase inhibitor that can promote VAIL; it is proposed that this occurs through increased V_1_V_O_ association (Hooper et al., 2022). This highlights the complexity of the relationship between V-ATPase inhibition and VAIL. A more specific V-ATPase-targeted inhibition of VAIL has been achieved through the *Salmonella* effector protein SopF (Xu et al., 2019; Fischer et al., 2020; Ulferts et al., 2021; Hooper et al., 2022). SopF is an ADP-ribosyltransferase that can ADP-ribosylate the subunit c of V_O_ of the V-ATPase complex (Xu et al., 2019; Xu et al., 2022). In doing so, the V-ATPase–ATG16L1 interaction is abolished, whereas V-ATPase activity appears to remain intact (Xu et al., 2019). SopF appears to have no effect on canonical autophagy, so can be used to distinguish canonical autophagy from VAIL.

## STIL induction and mechanisms

An alternative pathway that results in ATG8 conjugation to single membranes, termed STIL, was recently described. Here, the ATG8 conjugation complex consists of ATG5–ATG12 and TECPR1 in place of ATG16L1 (see poster). The critical step in STIL is the exposure of sphingomyelin to the cytosolic face of membranes (Boyle et al., 2023; Corkery et al., 2023; Kaur et al., 2023). Sphingomyelin is enriched in the plasma membrane, endocytic recycling compartment and trans-Golgi networks, and it is normally present only in the luminal leaflet of membranes (Ellison et al., 2020). Damage to the membrane of these vesicles can induce exposure of sphingomyelin from the luminal side of the membrane to the cytosol, resulting in TECPR1 recruitment (Boyle et al., 2023).

The N-terminal dysferlin (DysF) domain of TECPR1 directly binds sphingomyelin, thus recruiting the lipidation machinery to target membranes (Boyle et al., 2023; Corkery et al., 2023; Kaur et al., 2023). TECPR1 also contains a C-terminal DysF domain, which might contribute to membrane binding. Mutations in either DysF domain reduce the amount of LC3B (also known as MAP1LC3B) lipidation induced by L-leucyl-L-leucine methyl ester (LLOMe), and both domains are necessary for TECPR1 lipid binding *in vitro* (Kaur et al., 2023). However, deletion of the C-terminal DysF does not appear to affect TECPR1 recruitment to damaged lysosomes in cells (Corkery et al., 2023). When expressed alone, only the N-terminal DysF domain was found to accumulate at pathogen-containing vacuoles and damaged endosomes (Boyle et al., 2023). TECPR1 also contains a pleckstrin homology (PH) domain, which has been proposed to contribute to membrane interactions when the DysF domain is bound to sphingomyelin, although deletion of this PH domain does not seem to affect TECPR1 localisation to damaged lysosomal membranes (Corkery et al., 2023; Kaur et al., 2023).

Damage to membranes, and thus sphingomyelin exposure, can occur for vacuoles formed after *Salmonella* or *Shigella* infection, although the precise events that alter the sphingomyelin distribution at infected compartments are not fully understood (Ellison et al., 2020). Pharmacological induction of lysosomal damage also induces STIL; treatment with LLOMe or dilinoleylmethyl-4-dimethylamino-butyrate-containing nanoparticles induces relocalisation of TECPR1 to the lysosome and is associated with sphingomyelin exposure (Corkery et al., 2023; Kaur et al., 2023). Strong recruitment can also be observed with glycyl-L-phenylalanine-β-naphthylamide treatment. Interestingly, ionophores like monensin or nigericin, which also drive VAIL, can induce STIL following a longer treatment period (Corkery et al., 2023; Kaur et al., 2023). LLOMe has been reported to induce VAIL (Cross et al., 2023), as well as STIL, suggesting that VAIL and STIL might have synergistic effects.

Importantly, the recognition of sphingomyelin by TECPR1 appears to be upstream of the recognition of burst vesicles; STIL therefore targets only modestly damaged vesicles that retain a lumen (Boyle et al., 2023), rather than ruptured vesicles. The latter type will be cleared by canonical autophagy induced by detection of intraluminal glycans by lectins (Thurston et al., 2012; Boyle and Randow, 2013; Ravenhill et al., 2019). Therefore, like VAIL, STIL recognises early perturbations in membrane integrity.

Mutating key residues in the TECPR1 DysF domain can be used to selectively inhibit STIL (Boyle et al., 2023; Kaur et al., 2023). Additionally, overexpression of the sphingomyelin phosphodiesterase nSMase2 (also known as SMPD3) inhibits STIL by rapidly degrading cytosolic sphingomyelin (Kaur et al., 2023). The presence of ATG16L1 in VAIL-deficient cells affects the levels of STIL, and it has been suggested that this could reflect competition between ATG16L1 and TECPR1 for the common ATG12–ATG5 conjugate (Kaur et al., 2023).

## Biological functions of CASM

VAIL has close links with immune responses to intracellular damage, particularly during infection (Wang et al., 2022). Mice that lack the WD40 domain of ATG16L1 exhibit reduced survival during IAV infection (Wang et al., 2021) and inhibition of VAIL during invasive *Salmonella* infection increases bacterial propagation (Xu et al., 2019). Additionally, STING acting as a proton channel suggests an important link between innate immunity and VAIL (Fischer et al., 2020; Liu et al., 2023). However, the precise association remains obscure, as VAIL has been shown to be dispensable for downstream STING-induced cytokine signalling (Liu et al., 2023).

VAIL has also been linked to the regulation of the biogenesis of acidified compartments. Members of the MiT/TFE family of basic helix-loop-helix leucine zipper transcription factors, namely, transcription factor EB (TFEB) and transcription factor binding to IGHM enhancer 3 (TFE3), have been described as master regulators of lysosomal biogenesis and are key in the maintenance of acidified cellular compartments (Sardiello et al., 2009; Settembre et al., 2011). Recent studies have shown that VAIL can activate TFEB- and TFE3-mediated transcription following lysosomal damage, and this response regenerates and maintains lysosome numbers (Nakamura et al., 2020; Goodwin et al., 2021). GABARAP, through sequestration of the complex comprising folliculin (FLCN) and one of the folliculin-interacting proteins (FNIP), is crucial to the response. FLCN–FNIP activates the GTPase activity of the RagC–RagD heterodimer, which itself plays a role in regulating the phosphorylation state of TFEB (Goodwin et al., 2021). This function of VAIL neatly connects the recognition of proton gradient dissipation to the regeneration of acidic compartments.

In certain contexts, VAIL recruits leucine-rich repeat kinase 2 (LRRK2) (Bentley-DeSousa and Ferguson, 2023 preprint; Eguchi et al., 2024). LRRK2 is an important kinase that phosphorylates Rab GTPases in order to regulate lysosomal damage response pathways and maintain lysosome integrity (Steger et al., 2016; Herbst et al., 2020). LRRK2 variants are associated with Parkinson's disease, as well as inflammatory disorders such as Crohn's disease (Hui et al., 2018; Herrick and Tansey, 2021). Notably, a polymorphism in ATG16L1 (T300A) is strongly linked to Crohn's disease susceptibility (Hampe et al., 2007), suggesting that the role of ATG16L1 in this inflammatory disorder might be linked to VAIL rather than macroautophagy.

In the context of LAP, it has been proposed that VAIL regulates fusion of phagosomes with lysosomes (Romao et al., 2013; Wang et al., 2022). This can promote clearance of engulfed pathogens and has also been shown to regulate the presentation of exogenous antigen on major histocompatibility complex class-II (MHC-II) by regulating the rate of phagosome–lysosome fusion (Ma et al., 2012; Fletcher et al., 2018; Ligeon et al., 2021). MHC-II is expressed on professional antigen-presenting cells, like macrophages and dendritic cells, and presents small peptides generated by lysosomal degradation of extracellular content to CD4^+^ T-cells, thereby shaping antibody responses. Reports describe both prolongation and acceleration of phagosome–lysosome fusion by VAIL, suggesting that this function is highly contextual (Romao et al., 2013; Gluschko et al., 2018, 2022). Similarly, entosis is a LAP-like cell death pathway, which also appears to exploit VAIL-mediated lysosomal targeting (Overholtzer et al., 2007; Florey et al., 2011; Fletcher et al., 2018). Entosis is a live cell-in-cell uptake event that culminates in the lysosomal degradation of the internalised cell. Entotic cells are a common feature in multiple human cancers, and the pathway reportedly promotes both pro- and anti-tumorigenic effects; how VAIL contributes to these tumorigenic functions warrants further study (Florey et al., 2011; Durgan and Florey, 2018).

It will be interesting to revisit some of the previously described instances where CASM has been observed to assess potential contributions of STIL. For instance, in a recently described mechanism of antigen cross-presentation, extracellular antigen was proposed to cross into the cytosol from damaged, sphingomyelin-positive phagosomes (Canton et al., 2021). Like LAP-induced VAIL, this might point to a role for STIL in antigen presentation, albeit in a distinct context. STIL also appears to contribute to membrane damage responses, as TECPR1 contributes to lysosomal recovery after LLOMe-induced damage (Corkery et al., 2023). However, maximal inhibition of damage-induced lysosome recovery is only seen after deletion of both ATG16L1 and TECPR1. This suggests that STIL and VAIL converge on at least some common downstream function with redundancy between the two pathways.

## Conclusions and future perspectives

Evidence so far suggests that CASM is a form of danger signalling indicating early organelle damage (Durgan and Florey, 2022). VAIL appears to represent a response to the erroneous neutralisation of compartments, mediated by an interaction between ATG16L1 and the V-ATPase complex, whereas STIL detects erroneously situated sphingomyelin via TECPR1. Although STIL and VAIL are triggered by distinct mechanisms, much remains to be determined about the relationship between them, and with autophagy. Are they truly degradative pathways? Do STIL and VAIL serve complementary or redundant functions in the recognition of membrane damage? Do these pathways compete for substrates and machinery? Are they antagonised by similar or distinct mechanisms?

The connection with cellular damage responses is consistent with the links between CASM and cell-autonomous infection responses (Beale et al., 2014; Xu et al., 2019; Wang et al., 2021, 2022; Boyle et al., 2023). CASM is triggered by infection in numerous settings but no clear non-redundant role in cell autonomous immunity has been demonstrated. Although STING plays crucial roles in innate immunity, type I interferon signalling and the recognition of infection, the contribution of CASM to these immune functions remains elusive (Fischer et al., 2020; Liu et al., 2023). We hypothesise that CASM is likely degradative in function as this is often the fate of stressed membranes tagged with ubiquitin or ubiquitin-like modifiers. However, common tools used to stimulate CASM (ionophore drugs, infection with pathogens and LLOMe) perturb many cellular compartments simultaneously and inhibit lysosomal function. With current tools it might be difficult to observe the degradation that might occur if a single compartment became defective and tagged by CASM. Thus, the wider biological purposes of CASM so far remain opaque and await more precise tools for further elucidation.

## Poster

Poster

## Panel 1. Conjugation of ATG8s to single membranes

Panel 1. Conjugation of ATG8s to single membranes

## Panel 2. pH gradient dissipation during VAIL induction

Panel 2. pH gradient dissipation during VAIL induction

## Panel 3. Modulating VAIL

Panel 3. Modulating VAIL

## Panel 4. The ATG8 conjugation machinery

Panel 4. The ATG8 conjugation machinery

## Panel 5. TECPR1 membrane recognition

Panel 5. TECPR1 membrane recognition
