# Supplementary figures and images for: Conjugation of ATG8s to single membranes at a glance
Source: J Cell Sci. 2024 Aug 15;137(15):jcs261031. doi: 10.1242/jcs.261031 (PMC11361636; doi:10.1242/jcs.261031)

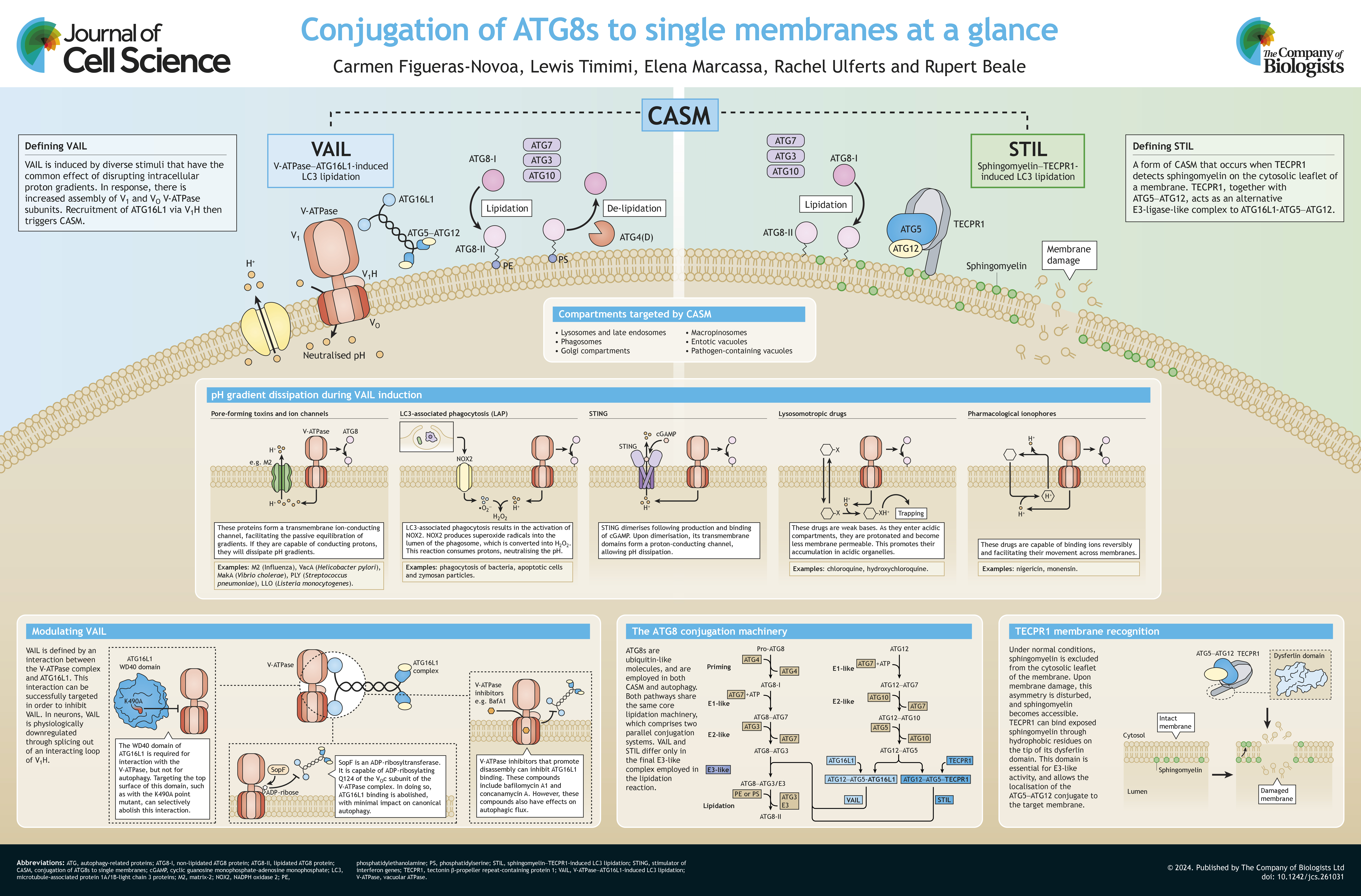

Supplement: Poster [file joces-137-261031-s1.jpg]

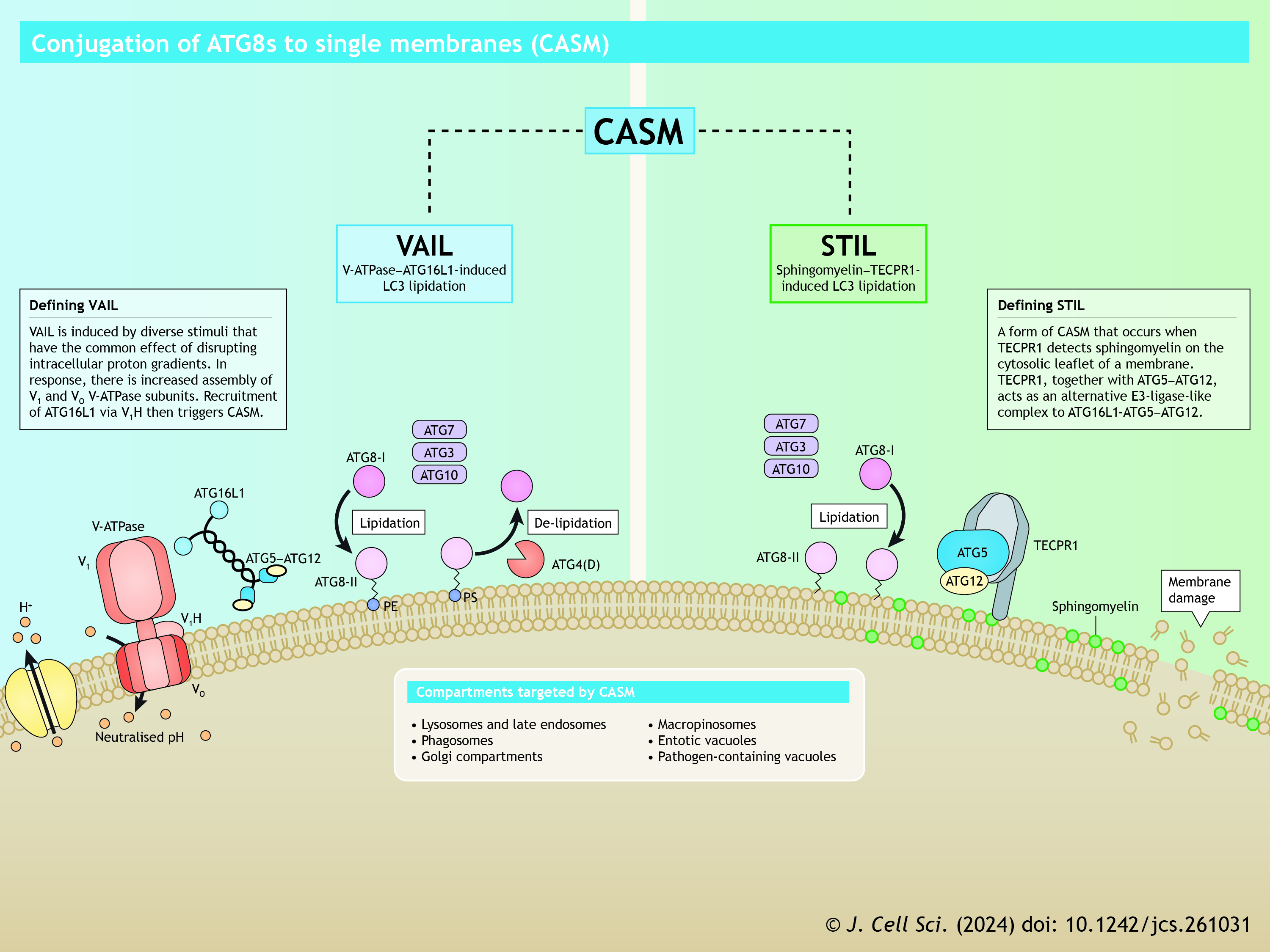

Supplement: Panel 1. Conjugation of ATG8s to single membranes [file joces-137-261031-s2.jpg]

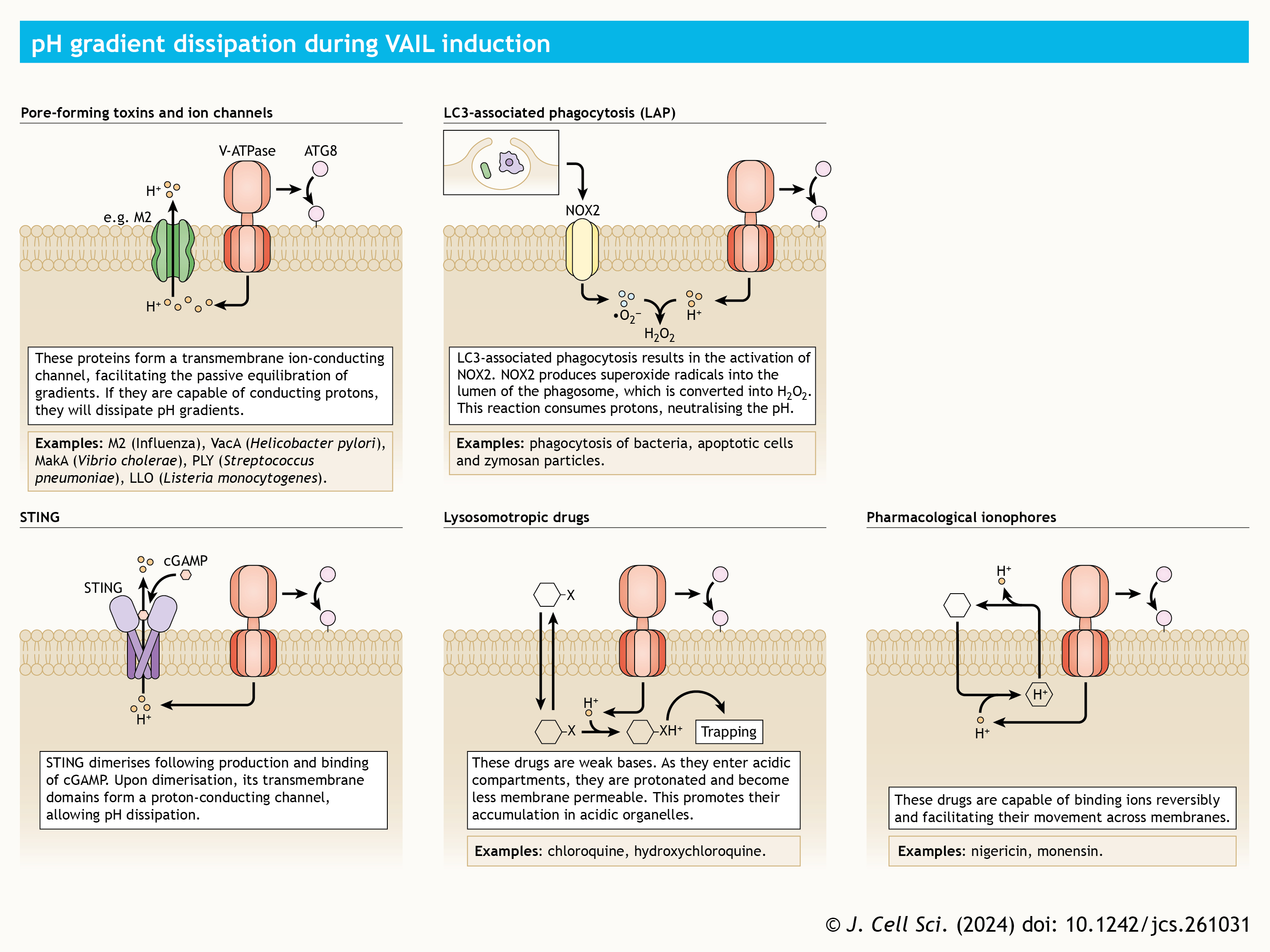

Supplement: Panel 2. pH gradient dissipation during VAIL induction [file joces-137-261031-s3.jpg]

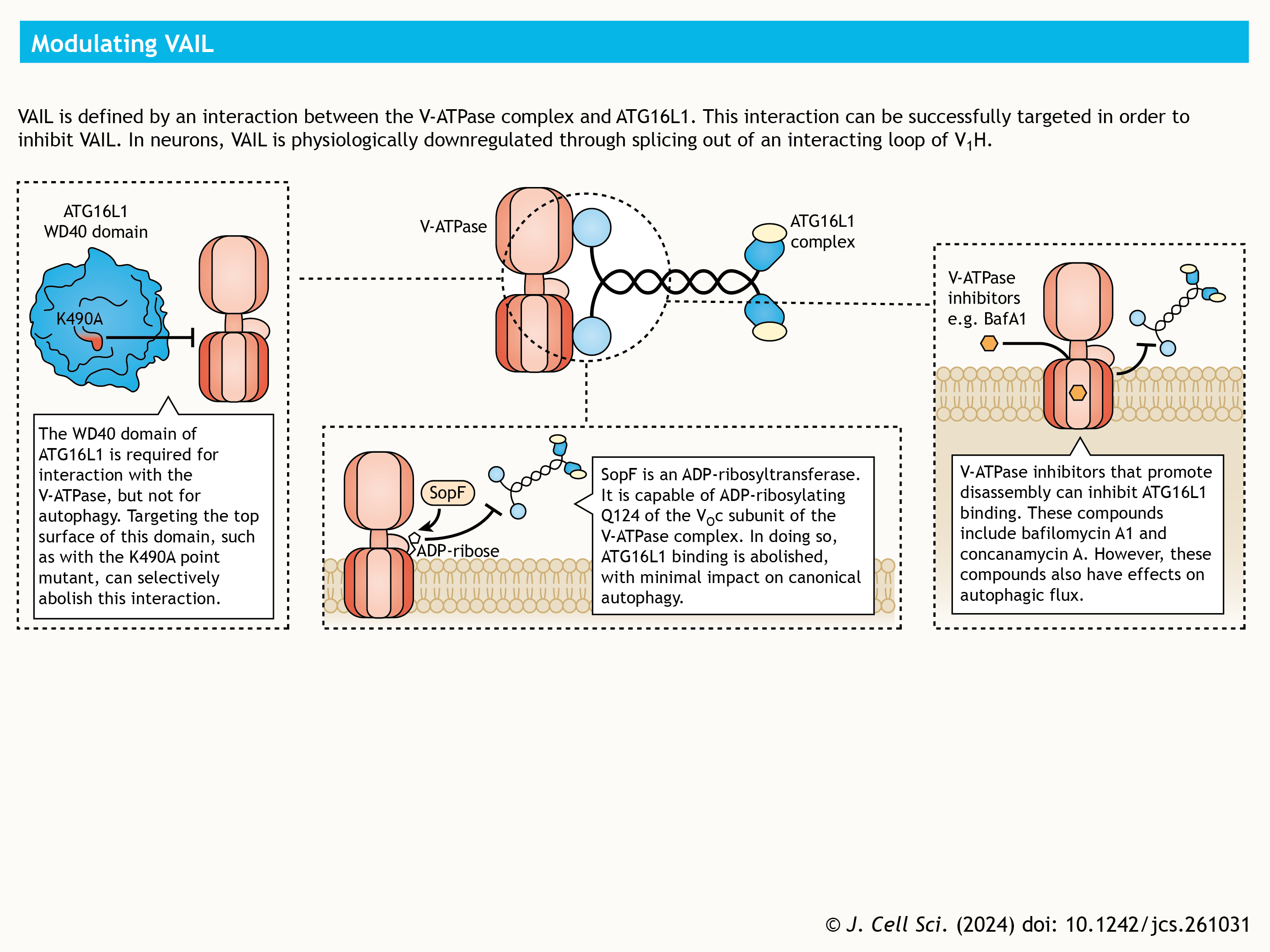

Supplement: Panel 3. Modulating VAIL [file joces-137-261031-s4.jpg]

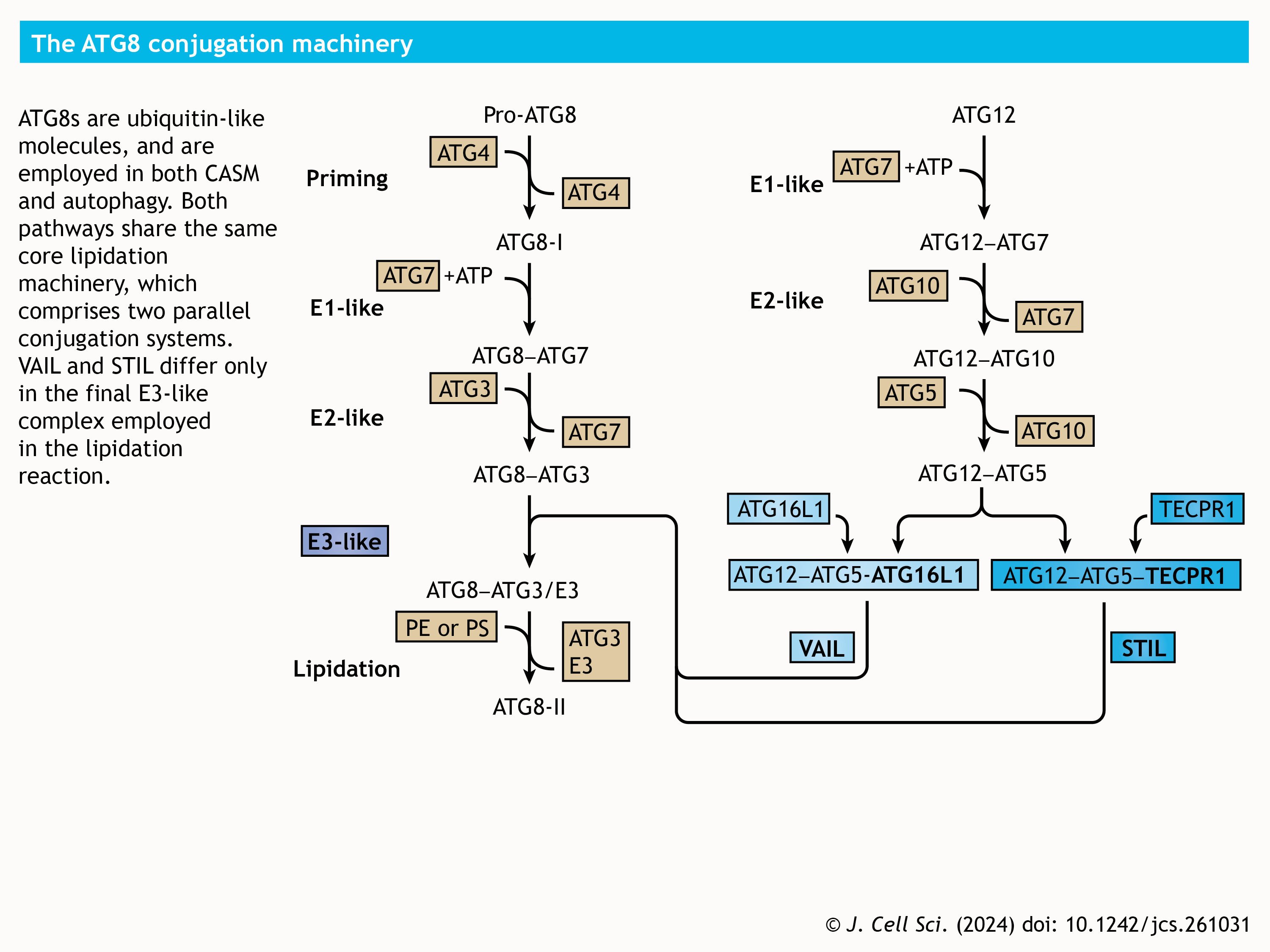

Supplement: Panel 4. The ATG8 conjugation machinery [file joces-137-261031-s5.jpg]

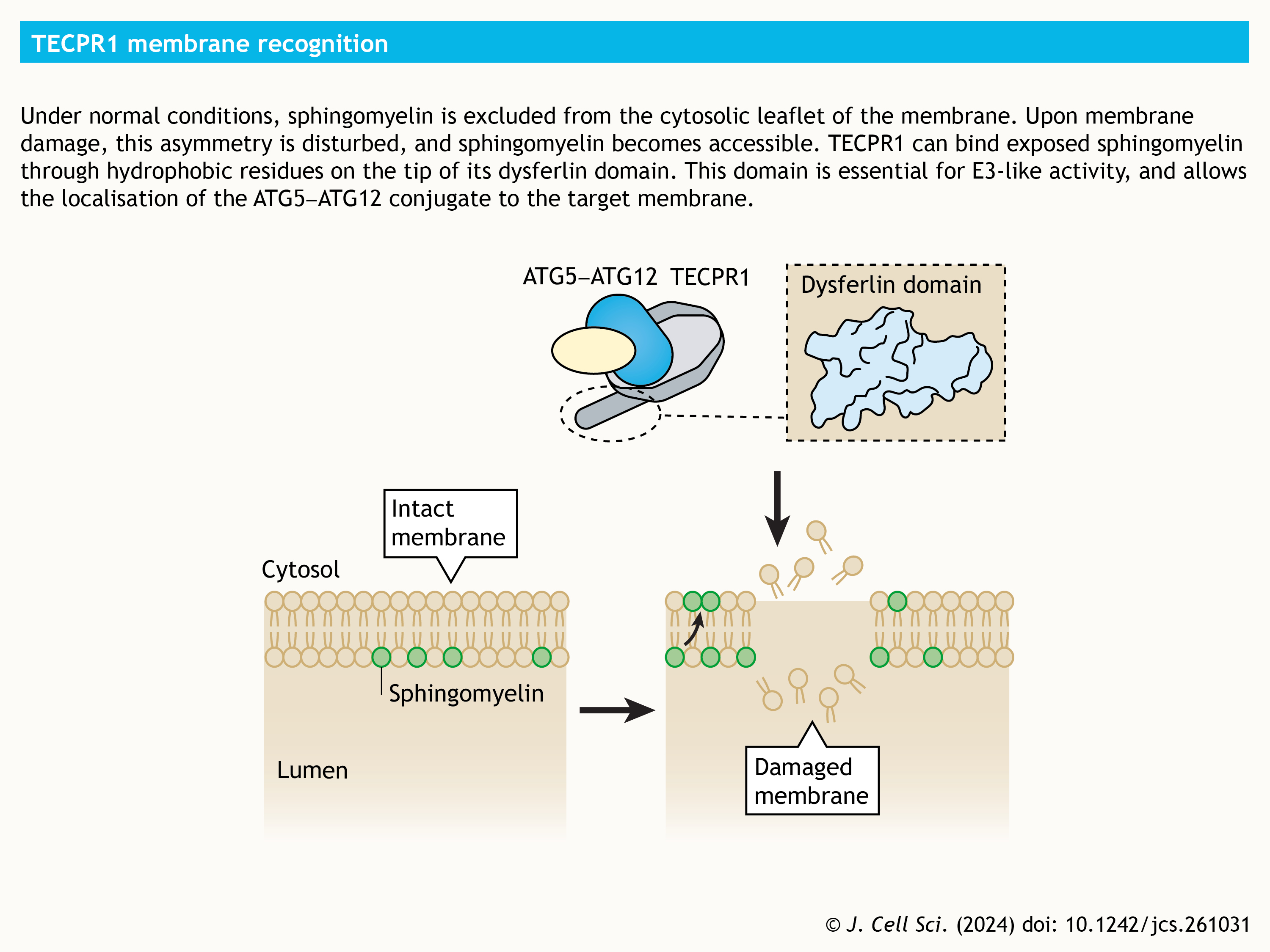

Supplement: Panel 5. TECPR1 membrane recognition [file joces-137-261031-s6.jpg]
